# Supplementary material for: End Sequence Analysis Toolkit (ESAT) expands the extractable information from single-cell RNA-seq data
Source: Genome Res. 2016 Oct;26(10):1397–410. doi: 10.1101/gr.207902.116 (PMC5052061; doi:10.1101/gr.207902.116)
Supplement: Supplemental Material [file supp_gr.207902.116_Supp_Materials.tar.gz › ESAT-esat_v0.1/doc/html/classbroad_1_1pda_1_1seq_1_1clip_1_1_pileup.html]

ESAT: broad.pda.seq.clip.Pileup Class Reference


ESAT


- Main Page
- Related Pages
- Packages
- Classes
- Files

- Class List
- Class Index
- Class Hierarchy
- Class Members

All Classes Namespaces Files Functions Variables Enumerator Pages

- broad
- pda
- seq
- clip
- Pileup

Public Member Functions |
Static Public Member Functions |
List of all members

broad.pda.seq.clip.Pileup Class Reference

Collaboration diagram for broad.pda.seq.clip.Pileup:

[legend]

|  |  |
| --- | --- |
| Public Member Functions | |
|  | Pileup (String bamFile, String annotationBedFile, String subregionBedFile, String genomeFasta, Collection< Predicate< Alignment >> readFilters) throws IOException |
|  | |
|  | Pileup (String bamFile, String fragmentEndWig, String annotationBedFile, String subregionBedFile, String genomeFasta, String regionOverlapperCountFile, String peakRegionBedFile, Collection< Predicate< Alignment >> readFilters) throws IOException |
|  | |
| Map< Annotation, Collection  < EmpiricalDistribution > > | getShuffledPositionsAllRegions (boolean fullyContained, Map< String, Double > nucProbs, int numPermutations) |
|  | |
| Map< Annotation, Double > | getScanPvalFragmentEndsMaxPositionAllRegionsBam (boolean fullyContained, Map< String, Double > nucProbs, int numPermutations, boolean fivePrimeEnd) |
|  | |
| Map< Integer, Double > | getScanPvalsFragmentEndPileupsWig (Annotation region, boolean fullyContained, Map< String, Double > nucProbs, int numPermutations) |
|  | |
| Map< Annotation, Map< Integer,   Double > > | getScanPvalsFragmentEndPileupsAllRegionsWig (boolean fullyContained, Map< String, Double > nucProbs, int numPermutations) |
|  | |
| void | writeScanPvalsFragmentEndPileupsAllRegionsWig (String outTable, String outBed, double pvalCutoffForBed, boolean fullyContained, Map< String, Double > nucProbs, int numPermutations) throws IOException |
|  | |
| double | getScanPvalFragmentEndsMaxPositionBam (Annotation region, boolean fullyContained, Map< String, Double > nucProbs, int numPermutations, boolean fivePrimeEnd) |
|  | |
| Collection< EmpiricalDistribution > | getShuffledPositions (Annotation region, boolean fullyContained, Map< String, Double > nucProbs, int numPermutations) |
|  | |
| EmpiricalDistribution | getFragmentEndsFromBamFile (Annotation region, boolean fullyContained, boolean fivePrimeEnd) |
|  | |
| Map< String, Collection< Gene > > | getAnnotation () |
|  | |

|  |  |
| --- | --- |
| Static Public Member Functions | |
| static void | main (String[] args) throws IOException |
|  | |

## Detailed Description

Author
:   prussell

## Constructor & Destructor Documentation

|  |  |  |  |
| --- | --- | --- | --- |
| broad.pda.seq.clip.Pileup.Pileup | ( | String | *bamFile*, |
|  |  | String | *annotationBedFile*, |
|  |  | String | *subregionBedFile*, |
|  |  | String | *genomeFasta*, |
|  |  | Collection< Predicate< Alignment >> | *readFilters* |
|  | ) |  | throws IOException |

Parameters
:   |  |  |
    | --- | --- |
    | bamFile | Bam file of alignments |
    | annotationBedFile | Bed transcriptome annotation |
    | genomeFasta | Fasta file of genome |
    | readFilters | Read filters to use |

Exceptions
:   |  |  |
    | --- | --- |
    | IOException |  |

Here is the caller graph for this function:

|  |  |  |  |
| --- | --- | --- | --- |
| broad.pda.seq.clip.Pileup.Pileup | ( | String | *bamFile*, |
|  |  | String | *fragmentEndWig*, |
|  |  | String | *annotationBedFile*, |
|  |  | String | *subregionBedFile*, |
|  |  | String | *genomeFasta*, |
|  |  | String | *regionOverlapperCountFile*, |
|  |  | String | *peakRegionBedFile*, |
|  |  | Collection< Predicate< Alignment >> | *readFilters* |
|  | ) |  | throws IOException |

Parameters
:   |  |  |
    | --- | --- |
    | bamFile | Bam file of alignments |
    | annotationBedFile | Bed transcriptome annotation |
    | genomeFasta | Fasta file of genome |
    | regionOverlapperCountFile | File of overlapper counts for regions (can be incomplete) |
    | peakRegionBedFile | Bed file of peak region (subregion of the parent gene enclosing all reads overlapping the region) corresponding to each region. Bed name is the same as region name. |
    | readFilters | Read filters to use |

Exceptions
:   |  |  |
    | --- | --- |
    | IOException |  |

## Member Function Documentation

|  |  |  |  |  |
| --- | --- | --- | --- | --- |
| Map<String, Collection<Gene> > broad.pda.seq.clip.Pileup.getAnnotation | ( |  | ) |  |

Get the transcriptome annotation

Returns
:   The annotation

|  |  |  |  |
| --- | --- | --- | --- |
| EmpiricalDistribution broad.pda.seq.clip.Pileup.getFragmentEndsFromBamFile | ( | Annotation | *region*, |
|  |  | boolean | *fullyContained*, |
|  |  | boolean | *fivePrimeEnd* |
|  | ) |  |  |

Get an empirical distribution storing the fragment ends of all fragments overlapping the region Fragment ends are in transcript coordinates with respect to the parent transcript

Parameters
:   |  |  |
    | --- | --- |
    | parent | Parent gene |
    | region | Region within parent to get pileup over |
    | fullyContained | Only include fragments that are fully contained in the subregion |
    | fivePrimeEnd | Report the 5' end of fragments (if false, reports 3' end) |

Returns
:   Distribution of fragment endpoints in transcript coordinates

Here is the caller graph for this function:

|  |  |  |  |
| --- | --- | --- | --- |
| Map<Annotation, Double> broad.pda.seq.clip.Pileup.getScanPvalFragmentEndsMaxPositionAllRegionsBam | ( | boolean | *fullyContained*, |
|  |  | Map< String, Double > | *nucProbs*, |
|  |  | int | *numPermutations*, |
|  |  | boolean | *fivePrimeEnd* |
|  | ) |  |  |

For each region, find the highest fragment end pileup from the bam file. Randomly permute the fragments in a biased way according to background nucleotide probabilities. Get a scan P value for the real highest pileup in the region.

Parameters
:   |  |  |
    | --- | --- |
    | fullyContained | When computing number of fragments and peak region, include fully contained fragments only |
    | nucProbs | Background nucleotide probabilities |
    | numPermutations | Number of permutations per region |
    | fivePrimeEnd | Use the 5' end of fragments (if false, use 3' end) |

Returns
:   Map of region to P value

Here is the call graph for this function:

|  |  |  |  |
| --- | --- | --- | --- |
| double broad.pda.seq.clip.Pileup.getScanPvalFragmentEndsMaxPositionBam | ( | Annotation | *region*, |
|  |  | boolean | *fullyContained*, |
|  |  | Map< String, Double > | *nucProbs*, |
|  |  | int | *numPermutations*, |
|  |  | boolean | *fivePrimeEnd* |
|  | ) |  |  |

Find the highest fragment end pileup in the region from the bam file. Randomly permute the fragments in a biased way according to background nucleotide probabilities. Get a scan P value for the real highest pileup in the region.

Parameters
:   |  |  |
    | --- | --- |
    | region | The region |
    | fullyContained | When computing number of fragments and peak region, include fully contained fragments only |
    | nucProbs | Background nucleotide probabilities |
    | numPermutations | Number of permutations per region |
    | fivePrimeEnd | Use the 5' end of fragments (if false, use 3' end) |

Returns
:   Map of region to P value

Here is the call graph for this function:

Here is the caller graph for this function:

|  |  |  |  |
| --- | --- | --- | --- |
| Map<Annotation, Map<Integer, Double> > broad.pda.seq.clip.Pileup.getScanPvalsFragmentEndPileupsAllRegionsWig | ( | boolean | *fullyContained*, |
|  |  | Map< String, Double > | *nucProbs*, |
|  |  | int | *numPermutations* |
|  | ) |  |  |

Randomly permute the fragments in a biased way according to background nucleotide probabilities. For each region get a scan P value for pileup height of each position in the region as read from wig file.

Parameters
:   |  |  |
    | --- | --- |
    | fullyContained | If computing peak regions from the bam file, include fully contained fragments only |
    | nucProbs | Background nucleotide probabilities |
    | numPermutations | Number of permutations per region |

Returns
:   Map of region to scan P value for each position in region

Here is the call graph for this function:

Here is the caller graph for this function:

|  |  |  |  |
| --- | --- | --- | --- |
| Map<Integer, Double> broad.pda.seq.clip.Pileup.getScanPvalsFragmentEndPileupsWig | ( | Annotation | *region*, |
|  |  | boolean | *fullyContained*, |
|  |  | Map< String, Double > | *nucProbs*, |
|  |  | int | *numPermutations* |
|  | ) |  |  |

Randomly permute the fragments in a biased way according to background nucleotide probabilities. Get a scan P value for pileup height of each position in the region as read from wig file.

Parameters
:   |  |  |
    | --- | --- |
    | region | The region |
    | fullyContained | If computing peak region from the bam file, include fully contained fragments only |
    | nucProbs | Background nucleotide probabilities |
    | numPermutations | Number of permutations |

Returns
:   Scan P value for each position in region

Here is the call graph for this function:

Here is the caller graph for this function:

|  |  |  |  |
| --- | --- | --- | --- |
| Collection<EmpiricalDistribution> broad.pda.seq.clip.Pileup.getShuffledPositions | ( | Annotation | *region*, |
|  |  | boolean | *fullyContained*, |
|  |  | Map< String, Double > | *nucProbs*, |
|  |  | int | *numPermutations* |
|  | ) |  |  |

Get shuffled sets of positions within the span covered by fragments overlapping the region Each set contains a number of positions equal to the number of fragments overlapping the region Random shuffled positions are biased by nucleotide

Parameters
:   |  |  |
    | --- | --- |
    | region | Region within parent gene |
    | fullyContained | When getting the span covered by fragments overlapping the region, include fully contained fragments only |
    | nucProbs | Background probabilities of each nucleotide; must add up to 1 |
    | numPermutations | Number of random sets to get |

Returns
:   Collection of random position sets; each set is presented in an empirical distribution

Here is the caller graph for this function:

|  |  |  |  |
| --- | --- | --- | --- |
| Map<Annotation, Collection<EmpiricalDistribution> > broad.pda.seq.clip.Pileup.getShuffledPositionsAllRegions | ( | boolean | *fullyContained*, |
|  |  | Map< String, Double > | *nucProbs*, |
|  |  | int | *numPermutations* |
|  | ) |  |  |

For each region get several shufflings of the positions

Parameters
:   |  |  |
    | --- | --- |
    | fullyContained | When computing number of fragments and peak region, include fully contained fragments only |
    | nucProbs | Background nucleotide probabilities |
    | numPermutations | Number of permutations per region |

Returns
:   Map of region to collection of random permutations

Here is the call graph for this function:

|  |  |  |  |  |  |
| --- | --- | --- | --- | --- | --- |
| static void broad.pda.seq.clip.Pileup.main | ( | String[] | *args* | ) | throws IOException |

static

Parameters
:   |  |  |
    | --- | --- |
    | args |  |

Exceptions
:   |  |  |
    | --- | --- |
    | IOException |  |

Here is the call graph for this function:

|  |  |  |  |
| --- | --- | --- | --- |
| void broad.pda.seq.clip.Pileup.writeScanPvalsFragmentEndPileupsAllRegionsWig | ( | String | *outTable*, |
|  |  | String | *outBed*, |
|  |  | double | *pvalCutoffForBed*, |
|  |  | boolean | *fullyContained*, |
|  |  | Map< String, Double > | *nucProbs*, |
|  |  | int | *numPermutations* |
|  | ) |  | throws IOException |

Randomly permute the fragments in a biased way according to background nucleotide probabilities. For each region get a scan P value for pileup height of each position in the region as read from wig file. Write information to a table.

Parameters
:   |  |  |
    | --- | --- |
    | outTable | Output table file |
    | outBed | Bed file for significant positions |
    | pvalCutoffForBed | P value cutoff for significant position |
    | fullyContained | If computing peak regions from the bam file, include fully contained fragments only |
    | nucProbs | Background nucleotide probabilities |
    | numPermutations | Number of permutations per region |

Returns
:   Map of region to scan P value for each position in region

Here is the call graph for this function:

---

The documentation for this class was generated from the following file:

- java/broad/pda/seq/clip/Pileup.java


---

Generated on Fri Aug 15 2014 15:11:01 for ESAT by  

 1.8.7
